# Supplementary material for: Nucleotide, Phospholipid, and Kynurenine Metabolites Are Robustly Associated with COVID-19 Severity and Time of Plasma Sample Collection in a Prospective Cohort Study
Source: Int J Mol Sci. 2023 Dec 26;25(1):346. doi: 10.3390/ijms25010346 (PMC10779247; doi:10.3390/ijms25010346)
Supplement: Supplementary file 1 [file ijms-25-00346-s001.zip › Supplementary Information_IJMS_revieweredits1.pdf]

Supplementary Information

Supplementary File S1. Metabolite literature review, related to Introduction and Discussion. Metabolites that mapped to pathways with significant associations with severity model sets (only pre-COVID-19; pre- and during COVID-19; pre-, during, and post-COVID-19) and/or time point model queries (transient or lingering effects). Relationships detected in the present study are listed, as well as those observed in previous studies for metabolites that have been previously reported. Metabolites without associated previous studies are novel to the present investigation. (file: "Supplementary_File_1_results_and_references.xlsx")

Supplementary File S2. Pathway cluster mappings of significant metabolites that mapped to pathways for severity and time of collection associations. Pathway mapping analysis using RaMP 2.0 for metabolites that were significant (FDR-adjusted p-value < 0.05) for (1) severity associations in (a) predisposition (only for the pre-COVID-19 time point (n = 441), (b) predisposition-and-acute (pre- and during (n = 86) COVID-19 time points), or (c) persistent (pre-, during, and post- (n = 82) COVID-19 time points) or (2) time associations as (a) transient (significantly different from pre- to during COVID-19 and during to post-COVID-19) or (b) lingering (significantly different from pre- to post-COVID-19) were mapped to pathways using RaMP 2.0. The first two tabs of the file contain cluster information, referenced in the "new_cluster" column of each results file, which was used to generate **Figures 3 and 4** of the manuscript.

Supplementary File S3. Predisposition metabolites associated with COVID-19 severity. Associations with ordinal COVID-19 severity for metabolites that are significantly associated with severity in only the pre-COVID-19 time point (n = 441, FDR adjusted p-value < 0.05), as modeled in **Equation 1, Methods**. (file: "Supplementary_Files_2_3_and_4_severity_model_results.xlsx" Sheet = Predisposition Metabolites)

Supplementary File S4. Predisposition-and-acute metabolites associated with COVID-19 severity. Associations with ordinal COVID-19 severity for metabolites that are significantly associated with severity in pre-COVID-19 (n = 441) and during COVID-19 (n = 86) time points with the same directionality of association (FDR adjusted p-value < 0.05), as modeled in **Equation 1, Methods**. (file: "Supplementary_Files_2_3_and_4_severity_model_results.xlsx" Sheet = Predisposition-and-Acute Metabs)

Supplementary File S5. Persistent metabolites associated with COVID-19 severity. Associations with ordinal COVID-19 severity for metabolites that are significantly associated with severity in pre-COVID-19 (n = 441), during (n = 86), and post-COVID-19 (n = 82) time points with the same directionality of association (FDR adjusted p-value < 0.05), as modeled in **Equation 1, Methods**. (file: "Supplementary_Files_2_3_and_4_severity_model_results.xlsx" Sheet = Persistent Metabolites)

Supplementary File S6. Transient metabolite changes during COVID-19. Associations with time point for metabolites that are transiently perturbed during COVID-19 infection. Estimate coefficients and standard errors of associations between metabolites and time point for

metabolites with FDR-adjusted p-values < 0.05 at pre-COVID-19 (n = 441) vs during (n = 86) COVID-19 and during COVID-19 vs post-COVID-19 (n = 82) but not pre-COVID-19 vs post-COVID-19, as modeled in **Equation 2**, **Methods**. (file: "Supplementary_Files_2_and_3_time_results.xlsx" Sheet = transient metabolites)

Supplementary File S7. Lingering metabolite changes after COVID-19. Associations with time point for metabolites that remain perturbed after COVID-19 infection. Estimate coefficients and standard errors of associations between metabolites and time point for metabolites with FDR-adjusted p-values < 0.05 at (a) during (n = 86) COVID-19 vs post-COVID-19 (n = 82) and pre-COVID-19 (n = 441) vs post-COVID-19 but not pre-COVID-19 vs during COVID-19, and (b) pre-COVID-19 vs during COVID-19 and pre-COVID-19 vs post-COVID-19 but not during COVID-19 vs post-COVID-19, as modeled in **Equation 2, Methods**. (file: "Supplementary_Files_1_and_2_time_results.xlsx" Sheet = lingering metabolites)

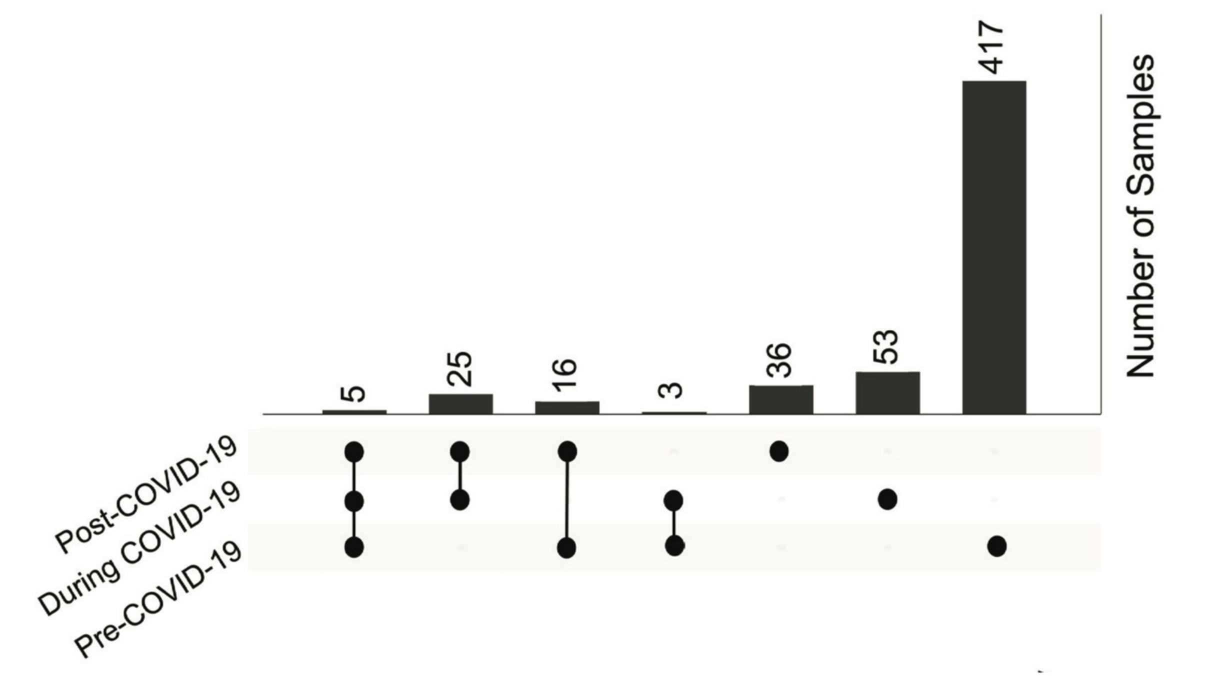

Supplementary Figure S1. Patient samples available at each time of sample collection. Shared sets reflect patients for which samples are available at multiple time points. Distinct sets reflect patients with samples available at only the respective time point.

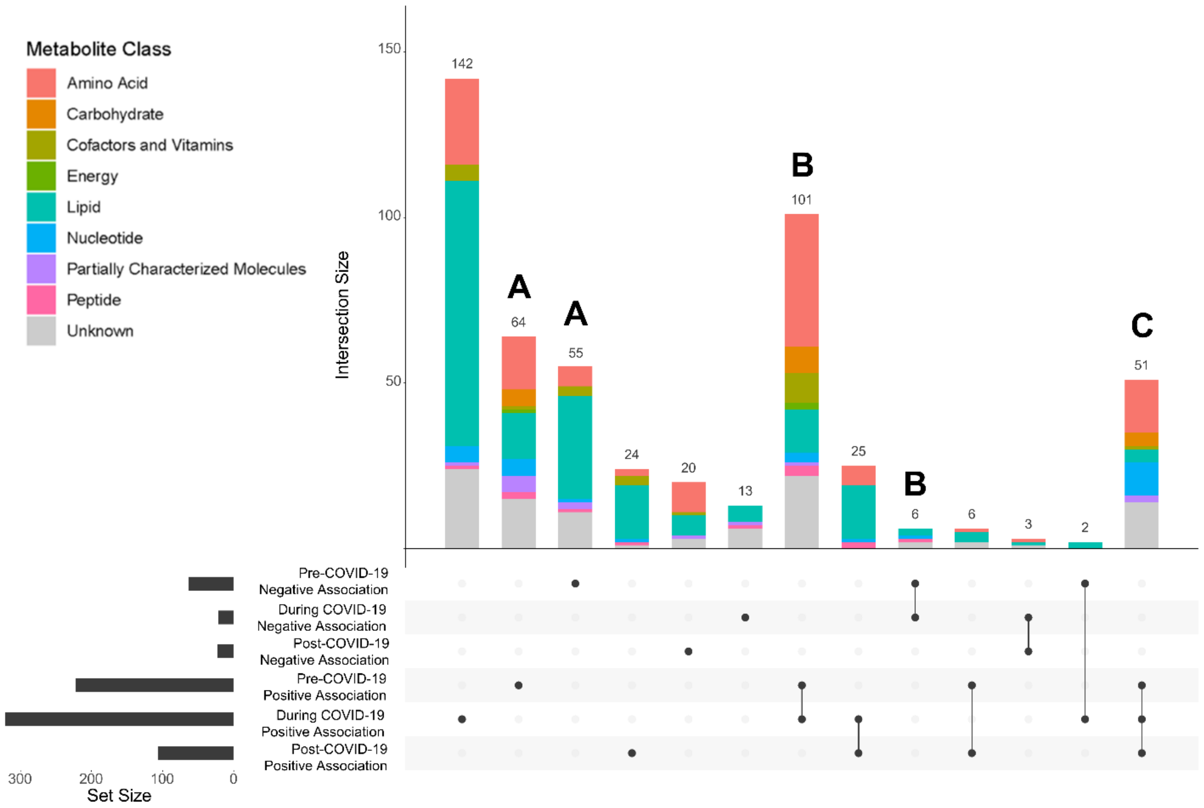

Supplementary Figure S2. Numbers of significant metabolites in all possible sets of severity model data. UpSet plot of metabolites significantly associated with COVID-19 severity at each time of sample collection (pre-, during, and/or post-COVID-19, FDR-adjusted p-value < 0.05). Annotations reflect the comparisons discussed: (A) predisposition metabolites uniquely associated with severity at only pre-COVID-19, (B) predisposition-and-acute metabolites associated with severity at both pre- and during COVID-19, and (C) persistent metabolites associated with severity at all times of sample collection—only positive associations were shared across all times of sample collection.

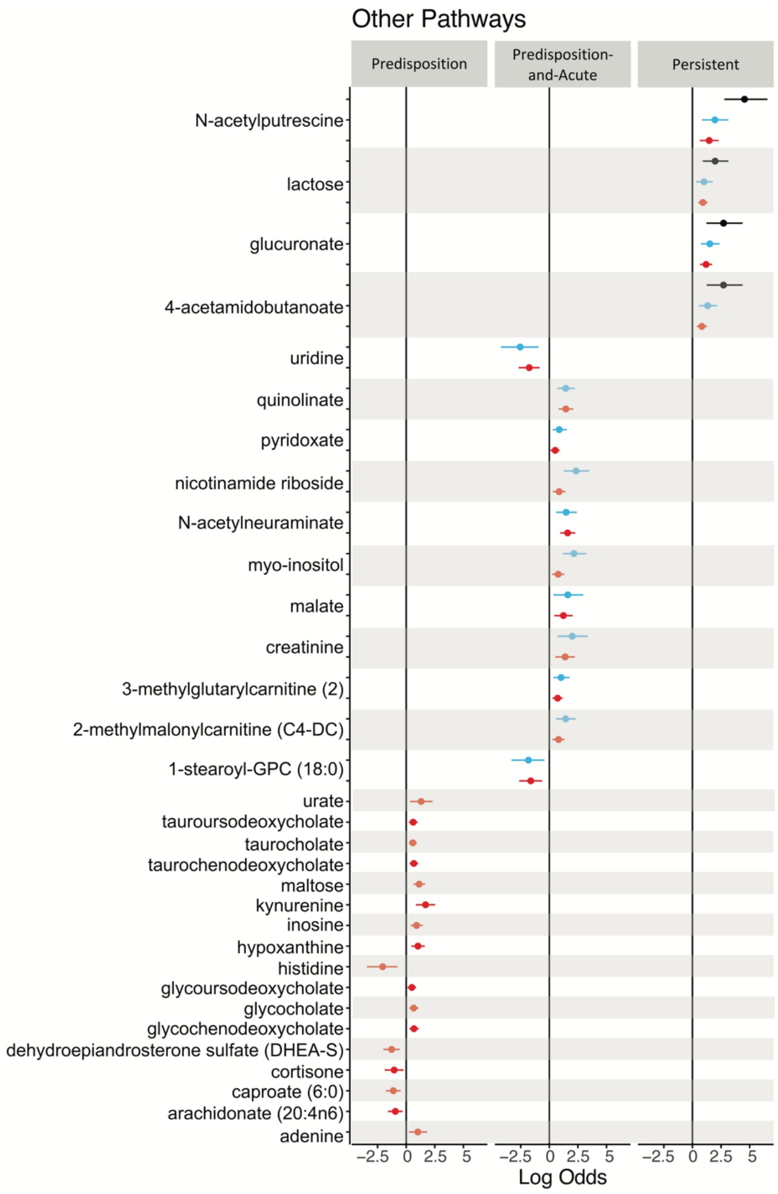

Supplementary Figure S3. Metabolites significantly associated with COVID-19 severity associations that map to unclustered biological pathways. Log odds of significant associations between metabolites and COVID-19 severity (**Equation 1**) for metabolites belonging to pathways that did not cluster with others. Sets of interest include: predisposition metabolites (only significantly associated with severity (FDR-adjusted p-values < 0.05) in pre-COVID-19 samples (n = 441)) (see **Supplementary File 3**); predisposition-and-acute metabolites (significantly associated with severity (FDR-adjusted p-values < 0.05) in pre- (n = 441) and during (n = 86) COVID-19 samples) (see **Supplementary File 4**); and persistent metabolites (significantly associated with severity (FDR-adjusted p-values < 0.05) in all samples (n = 441, 86, and 82 for pre-, during, and post-COVID-19 samples, respectively) (see **Supplementary File 5**).

Supplementary Table S1. Sensitivity analysis testing the effects of individual comorbidities in the severity models, one at a time. Metabolite sets (predisposition, predisposition-and-acute, and persistent) significantly associated with severity (**Equation 1**) were compared between the two following models: 1) the original composite Charlson Index covariable, and 2) each comorbidity used as an individual covariable (binary Yes/No for presence at COVID-19 diagnosis for each). The cells indicate the number of significant metabolites that overlapped between both models with the percentage of overlap relative to the original model in parentheses.

Comorbidity type	Predisposition	Predisposition-and-Acute	Persistent
**Charlson Index**	119	107	51
**Stroke**	110 (92.44%)	94 (87.85%)	50 (98.04%)
**Type 2 Diabetes**	93 (78.15%)	83 (77.57%)	51 (100%)
**Cardiovascular Disease**	97 (81.51%)	87 (81.31%)	7 (13.73%)
**Chronic Obstructive Pulmonary Disease**	93 (78.15%)	78 (72.90%)	51 (100%)
**Major Depression**	96 (80.67%)	82 (76.64%)	51 (100%)
**Cancer**	97 (81.51%)	89 (83.18%)	51 (100%)

Supplementary Table S2. Days before or after COVID-19 diagnosis (mean and standard deviation) for patients included in the pre- (before diagnosis), during (within 28 days from diagnosis) or post-COVID-19 (at least 28 days after diagnosis) times of sample collection.

	Time Point			
	**Pre-COVID-19 (n = 441)**	**During COVID-19 (n = 86)**	**Post-COVID-19 (n = 82)**	**All Time Points (n = 609)**
**Days from COVID-19 Diagnosis (Mean (SD))**				
0	-1360 (764)	9 (7)	99 (28)	-1160 (869)
1	-1490 (879)	5 (3)	78 (30)	-839 (1000)
2	-1630 (699)	8 (8)	72 (41)	-672 (940)
3	-1770 (920)	6 (3)	80 (40)	-1160 (1140)
Total Mean	-1440 (803)	6 (5)	88 (34)	-1030 (953)

Supplementary Table S3. Comorbidity categories and associated weights used to calculate Charlson Index, adapted from (1). Associated ICD-10 codes for the comorbidities are available in the original publication.

Comorbidity Category	Weight
Myocardial infarction	1

Congestive heart failure	1
Peripheral vascular disease	1
Cerebrovascular disease	1
Dementia	1
Rheumatologic disease	1
Chronic pulmonary disease	1
Peptic ulcer disease	1
Mild liver disease	1
Diabetes (mild to moderate)	1
Diabetes with chronic complications	2
Hemiplegia or paraplegia	2
Renal disease	2
Any malignancy, including lymphoma and leukemia	2
Moderate or severe liver disease	3
Metastatic solid tumor	6
AIDS	6

Reference

1. Sundararajan V, Henderson T Fau - Perry C, Perry C Fau - Muggivan A, Muggivan A Fau - Quan H, Quan H Fau - Ghali WA, Ghali WA. New ICD-10 version of the Charlson comorbidity index predicted in-hospital mortality. Journal of Clinical Epidemiology. 2004;57(12):1288-94.
